# Rapidly Progressive Acute Liver Failure in Relapsed Multiple Myeloma

**DOI:** 10.7759/cureus.12346

**Published:** 2020-12-28

**Authors:** Anshu Wadehra, Bhavin Chokshi, Connor R Buechler, Manmeet M Singh

**Affiliations:** 1 Internal Medicine, Wayne State University/Detroit Medical Center, Detroit, USA

**Keywords:** acute liver failure, multiple myeloma

## Abstract

Multiple myeloma affects upwards of 30,000 people every year and has significant morbidity and mortality. Common complications of the disease involve lytic bone lesions, hypercalcemia, anemia, and acute renal failure. A rare, yet serious, complication includes acute liver failure secondary to hepatic plasma cell infiltration. While this is reported seldom in living patients, it is found in upwards of 40% of patients incidentally on imaging or during autopsy. Conscientious and meticulous monitoring of liver function tests allows for early detection of liver failure in multiple myeloma; thus, allowing for broader therapeutic options overall.

## Introduction

Multiple myeloma is a neoplastic proliferation of plasma cells which produce monoclonal immunoglobulins [1]. Patients typically present with bone pain secondary to lytic lesions, hypercalcemia, significant anemia, and acute renal failure [1]. A rare, but significant, complication of multiple myeloma includes the diagnosis of GI system involvement, particularly the liver. Although plasma cell infiltration is not uncommonly seen during autopsy of patients with multiple myeloma, it is very rarely detected in living patients [2]. In patients with liver involvement due to multiple myeloma, the typical presentation is liver failure, significant hyperbilirubinemia, and rapid deterioration [3]. Additionally, the patients with liver involvement typically have a very poor prognosis [3]. We present a case of an elderly gentleman presenting with rapidly progressive acute liver failure secondary to atypical plasma cell infiltration of the liver. We want to add to the current understanding of this entity by underscoring the clinical features, diagnostics, therapeutic options, and effect on prognosis.

## Case presentation

The patient was a 79-year-old male with a history of immunoglobulin G (IgG) kappa multiple myeloma, along with multiple other medical comorbidities. Initial treatment of the disease involved lenalidomide, dexamethasone, along with autologous bone marrow transplant. His multiple myeloma progressed despite multiple lines of treatment. Given his disease progression, he was further treated with pomalidomide, dexamethasone, and ixazomib.

The patient presented to the hospital for recurrent dizziness episodes and failure to thrive. Subtle confusion was noted on examination. On admission, laboratory workup showed a significant direct hyperbilirubinemia with total bilirubin of 5.73 mg/dL and direct bilirubin of 3.53 mg/dL. However, alanine aminotransferase (ALT), aspartate aminotransferase (AST), and alkaline phosphatase levels were within normal limits. Of note, one month prior to admission, routine laboratory work showed a mildly elevated total bilirubin of 1.66 mg/dL. Synthetic function of the liver yielded an albumin level of 2.1 g/dL and international normalized ratio (INR) of 3.30 with partial thromboplastin time of 90.8 seconds that was significantly elevated from laboratory workup done two weeks prior to admission, which showed an INR of 1.52. In addition, clotting factor activity was significant for decreased factor IX, XI, XII activity, further indicating decreased liver synthetic activity. IgG was markedly elevated at 3,153 mg/dL. Additionally, viral hepatitis and autoimmune panel were negative.

**Figure 1:**
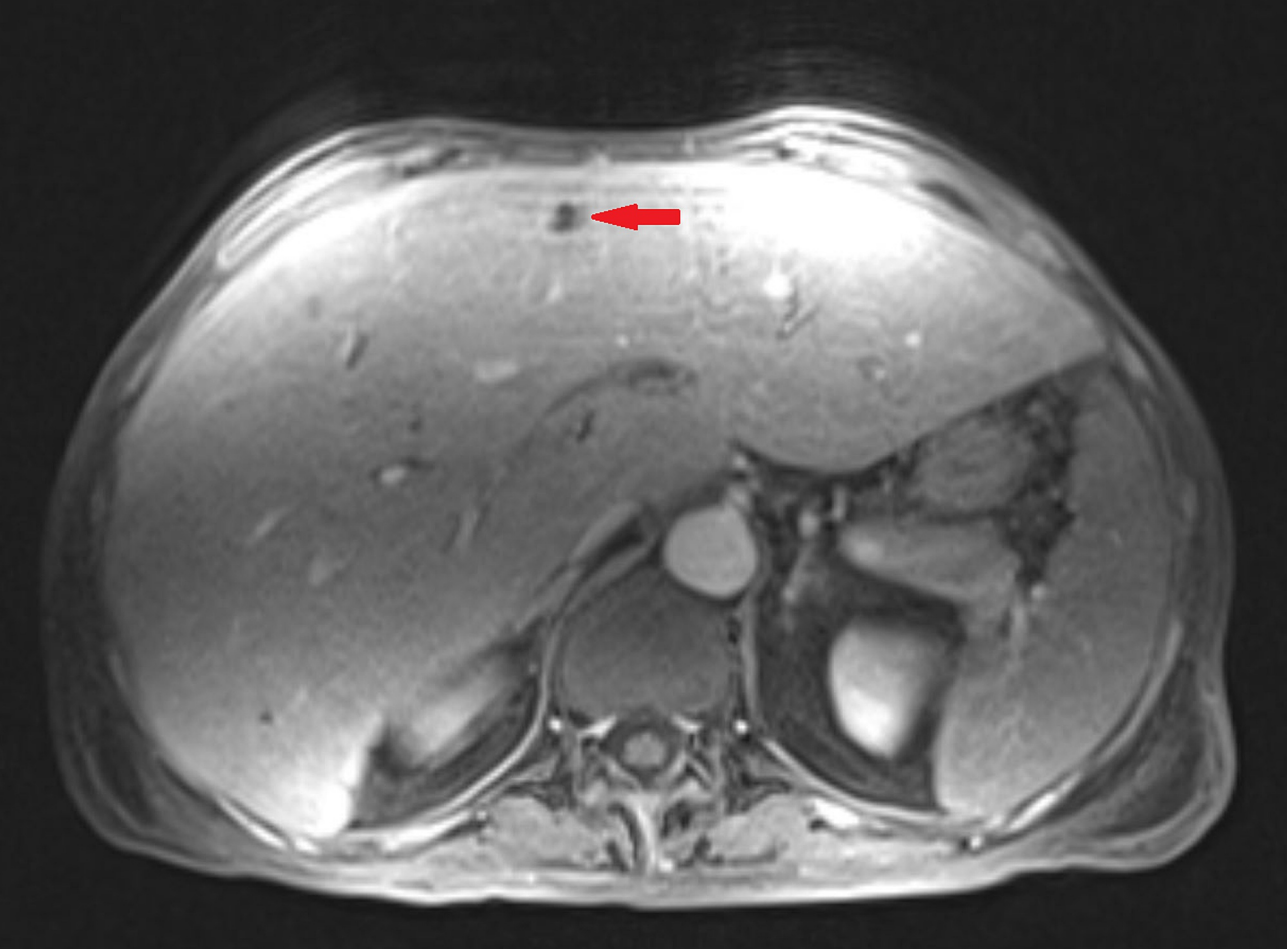
MRI of the liver showing a nodular hepatic lesion (red arrow).

**Figure 2:**
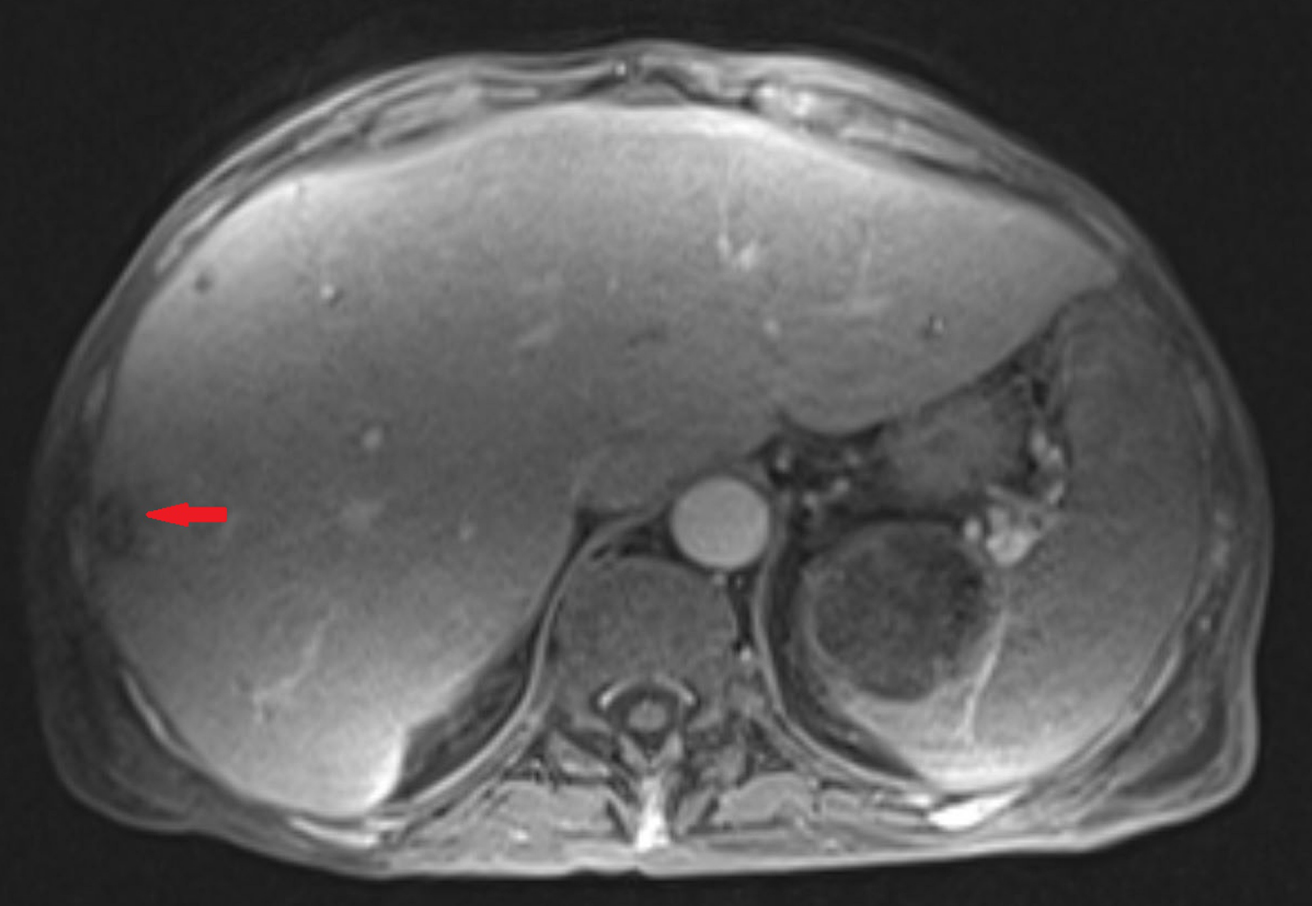
MRI of the liver showing a nodular hepatic lesion (red arrow).

Further workup for the patient’s liver function test derangements included MRI of the liver, which was significant for hepatomegaly with multiple new nodular hepatic masses, measuring as large as 1.6 cm (Figures 1, 2). In addition, areas of metastatic disease to the right iliac bone as well as a splenic mass were appreciated. Transjugular liver biopsy showed diffuse infiltration by atypical plasma cells within the liver parenchyma. The atypical plasma cells contained both Dutcher and Russell bodies and were positive for CD138 on immunohistochemistry analysis with kappa restriction on in situ hybridization.

During his hospital course, patient’s bilirubin continued to increase with a peak level of 11.24 mg/dL, with a direct bilirubin of 7.11 mg/dL and relatively normal values of ALT, AST, and alkaline phosphatase. He was given multiple units of fresh frozen plasma to correct his underlying coagulopathy. He exhibited some mild encephalopathy which responded to lactulose. Oncology tumor board discussion led to the decision to attempt pulse dose dexamethasone 40 mg for four days. Upon completion of this course, only minimal improvement of the patient’s symptoms and disease burden were noted. The patient was no longer a candidate for further chemotherapy or other therapeutic options. He elected for hospice care and passed away shortly thereafter.

## Discussion

Plasma cell infiltration within the liver has been reported in up to 40% cases of multiple myeloma; however, this is only discovered incidentally on imaging or during autopsy and is rarely discovered in living patients [2,3]. The prevalence among living patients is estimated at around 0.4% [3], which makes it a significantly rare entity in multiple myeloma patients. It is uncommon for patients to have acute liver failure as an initial presentation of multiple myeloma and is more commonly seen in patients with relapsed disease [4]. The mechanism of liver failure in this patient group involves either direct invasion by plasma cells, plasmacytomas, light chain deposition, or amyloid deposition [5]. Patients present with abnormal liver function tests, particularly with hyperbilirubinemia, and typically near-normal AST and ALT levels. Transaminitis has been rarely cited in previous literature [6]. As was the case in our patient, there was significant elevation of total and direct bilirubin, however, only mild elevations of ALT and AST were noted, which were 72 IU/L and 65 IU/L, respectively, at their peak during his hospitalization course.

Differential diagnosis of patients who present with conjugated hyperbilirubinemia without significant elevation of ALT and AST, in the absence of histology, can include drug-induced liver injury (DILI), nodular regenerative hyperplasia (NRH), and hepatic veno-occlusive disease (VOD)/sinusoidal obstruction syndrome (SOS). Enzyme abnormalities of NRH can present similarly to hepatic involvement of multiple myeloma, with rare elevation of alkaline phosphatase and mild elevations of AST and ALT; however, the clinical picture of NRH is dominated by portal hypertension and its sequelae, including ascites and variceal bleeding [7]. Isolated conjugated hyperbilirubinemia can also occur in congenital disorders such as Dubin-Johnson syndrome and Rotor syndrome, though non-overlapping epidemiological characteristics make these diseases easy to distinguish from myeloma involvement of the liver, as they typically appear in younger patients [8].

Careful attention to histology can assist in the differentiation of hepatic involvement of multiple myeloma, NRH, DILI, and VOD/SOS. The diagnostic feature of multiple myeloma is the presence of myeloma cells on biopsy. Though definitive identification of myeloma cells on hematoxylin/eosin stain can be difficult, immunohistochemical staining for CD138 is highly specific for plasma cells [9]. Myeloma cells often include intracytoplasmic inclusions of immunoglobulin light chains known as Dutcher bodies, which overlay the nucleus, or Russell bodies, which do not [10]. In-situ hybridization, rather than immunohistochemical staining, is recommended for proving restriction of immunoglobulin light chains due to the background presence of such chains outside the cells [9]. Though we did not perform flow cytometry analysis, plasma cell staining for CD56 is diagnostic of multiple myeloma.

The most common pattern of hepatic infiltration in multiple myeloma consists of diffuse sinusoidal infiltrates of plasma cells or nodules [11]. When nodules are present, NRH will show smaller nodules up to only 3 mm in diameter that lack myeloma cells and compress the surrounding architecture, including hepatic parenchyma as well as the portal and central veins [7]. VOD/SOS will present with characteristic occlusive lesions due to subintimal thickening of the central veins accompanied by sinusoidal dilation and centrilobular necrosis, but without evidence of plasma cells [12]. As DILI can present in various ways, it is a diagnosis of exclusion that should be entertained on the differential unless an alternative diagnosis becomes more likely. In our case, the preexisting multiple myeloma as well as characteristic microscopy, staining, and in situ hybridization proved convincing. Other common findings on microscopy, such as amyloidosis, hemosiderosis, and steatosis, are common in other neoplastic diseases and should not be thought of as specific to multiple myeloma.

Patients who develop extramedullary disease have a significantly poor prognosis [13]. Treatment is also difficult given the limited number of chemotherapy agents that can be administered, as many chemotherapeutic drugs lead to hepatotoxicity themselves. Common therapeutic options for newly diagnosed multiple myeloma include proteasome inhibitors, thalidomide, lenalidomide, cyclophosphamide, dexamethasone, and anthracyclines [14]. Our patient was treated specifically with lenalidomide, dexamethasone, pomalidomide, and ixazomib. He was further treated with pulse dose steroids as well during his admission. While there have been some reports of high-dose steroids effectively managing acute liver failure [15], in our patient this regimen did not help.

## Conclusions

Overall, liver involvement in multiple myeloma is a rare phenomenon seen in living patients. Upon our further review of the patient’s case, the fact that he had mildly increased bilirubin and INR approximately one month prior to admission should have warranted further work up of possible liver involvement at that time. Therefore, it is important to be cognizant and proactive about subtle liver function test derangements in multiple myeloma patients, as this can be an early sign of impending liver failure. Prompt diagnosis will allow for broader therapeutic options and overall more favorable outcomes.
